# Explainable Machine Learning Classification to Identify Vulnerable Groups Among Parenting Mothers: Web-Based Cross-Sectional Questionnaire Study

**DOI:** 10.2196/47372

**Published:** 2024-02-07

**Authors:** Akiko Hanai, Tetsuo Ishikawa, Shoko Sugao, Makoto Fujii, Kei Hirai, Hiroko Watanabe, Masayo Matsuzaki, Goji Nakamoto, Toshihiro Takeda, Yasuji Kitabatake, Yuichi Itoh, Masayuki Endo, Tadashi Kimura, Eiryo Kawakami

**Affiliations:** 1 Medical Data Mathematical Reasoning Team Advanced Data Science Project RIKEN Information R&D and Strategy Headquarters, RIKEN Yokohama Japan; 2 Artificial Intelligence Medicine Graduate School of Medicine Chiba University Chiba Japan; 3 Institute for Datability Science Osaka University Suita Japan; 4 Department of Extended Intelligence for Medicine The Ishii-Ishibashi Laboratory Keio University School of Medicine Tokyo Japan; 5 Graduate School of Human Sciences Osaka University Suita Japan; 6 Division of Health Sciences Graduate School of Medicine Osaka University Suita Japan; 7 Department of Reproductive Health Nursing Graduate School of Health Care Sciences Tokyo Medical and Dental University Tokyo Japan; 8 Department of Medical Informatics Graduate School of Medicine Osaka University Suita Japan; 9 Department of Pediatrics Graduate School of Medicine Osaka University Suita Japan; 10 Department of Integrated Information Technology College of Science and Engineering Aoyama Gakuin University Sagamihara Japan; 11 Department of Obstetrics and Gynecology Osaka University Graduate School of Medicine Suita Japan

**Keywords:** explainable machine-learning, unsupervised clustering, perceived support, resilience, adaptation, mother’s health, mobile phone, machine learning, web-based, parenting, postpartum, antenatal, survey, mother, women, newborn, psychosocial, infant, parents, children, depression, digital health, maternal

## Abstract

**Background:**

One life event that requires extensive resilience and adaptation is parenting. However, resilience and perceived support in child-rearing vary, making the real-world situation unclear, even with postpartum checkups.

**Objective:**

This study aimed to explore the psychosocial status of mothers during the child-rearing period from newborn to toddler, with a classifier based on data on the resilience and adaptation characteristics of mothers with newborns.

**Methods:**

A web-based cross-sectional survey was conducted. Mothers with newborns aged approximately 1 month (newborn cohort) were analyzed to construct an explainable machine learning classifier to stratify parenting-related resilience and adaptation characteristics and identify vulnerable populations. Explainable k-means clustering was used because of its high explanatory power and applicability. The classifier was applied to mothers with infants aged 2 months to 1 year (infant cohort) and mothers with toddlers aged >1 year to 2 years (toddler cohort). Psychosocial status, including depressed mood assessed by the Edinburgh Postnatal Depression Scale (EPDS), bonding assessed by the Postpartum Bonding Questionnaire (PBQ), and sleep quality assessed by the Pittsburgh Sleep Quality Index (PSQI) between the classified groups, was compared.

**Results:**

A total of 1559 participants completed the survey. They were split into 3 cohorts, comprising populations of various characteristics, including parenting difficulties and psychosocial measures. The classifier, which stratified participants into 5 groups, was generated from the self-reported scores of resilience and adaptation in the newborn cohort (n=310). The classifier identified that the group with the greatest difficulties in resilience and adaptation to a child’s temperament and perceived support had higher incidences of problems with depressed mood (relative prevalence [RP] 5.87, 95% CI 2.77-12.45), bonding (RP 5.38, 95% CI 2.53-11.45), and sleep quality (RP 1.70, 95% CI 1.20-2.40) compared to the group with no difficulties in perceived support. In the infant cohort (n=619) and toddler cohort (n=461), the stratified group with the greatest difficulties had higher incidences of problems with depressed mood (RP 9.05, 95% CI 4.36-18.80 and RP 4.63, 95% CI 2.38-9.02, respectively), bonding (RP 1.63, 95% CI 1.29-2.06 and RP 3.19, 95% CI 2.03-5.01, respectively), and sleep quality (RP 8.09, 95% CI 4.62-16.37 and RP 1.72, 95% CI 1.23-2.42, respectively) compared to the group with no difficulties.

**Conclusions:**

The classifier, based on a combination of resilience and adaptation to the child’s temperament and perceived support, was able identify psychosocial vulnerable groups in the newborn cohort, the start-up stage of childcare. Psychosocially vulnerable groups were also identified in qualitatively different infant and toddler cohorts, depending on their classifier. The vulnerable group identified in the infant cohort showed particularly high RP for depressed mood and poor sleep quality.

## Introduction

One life event that requires extensive resilience and adaptation is parenting. However, parenting difficulties have become highly varied and complex, especially for mothers, who are increasingly expected to balance their family and social roles [1,2]. The dynamics of parenting are further complicated by the rise of working parents, leading to evolving parenting environments and an amplified demand for social support [3]. The challenges of parenting have been compounded by the SARS-CoV-2 pandemic, which brought about blockades, physical distancing, and social isolation, adversely impacting mental health. These challenges are exacerbated by factors such as economic insecurity and the increased burden of childcare and housework [4].

A meta-analysis shows that 19.2% of women experience a major depressive episode during the first 3 months post partum, with most of these episodes occurring after delivery [5]. For this reason, most medical institutions in Japan evaluate the mother’s mental status during the 1-month health examination. Common risk factors of postpartum depression were high life stress, the lack of social support, current or past abuse, prenatal depression, and marital or partner dissatisfaction, which are not only limited to the early postpartum child-rearing periods [6]. Psychosocial stressors during long child-rearing periods are even more varied and complex, and individual mother’s personalities and cognitive characteristics must be comprehensively considered to support safe child-rearing practices [7,8]. Hence, we developed the Comprehensive Scale for Parenting Resilience and Adaptation (CPRA) to assess parenting difficulties based on environmental factors, maternal personality traits, and a mother’s perception of her child [2]. Thus, the CPRA could be used to evaluate the complexities of parenting difficulties.

Timely access to face-to-face social support has proven to be psychologically and socially challenging for mothers who struggle with parenting [9]. Studies have indicated that prior parenting knowledge and experience lead to resilience, as primiparous women have significantly lower resilience to perinatal depressive mood than multiparous women [10,11]. A web-based platform could be easily accessible for busy and vulnerable mothers. Thus, a web-based assessment and timely intervention could provide parenting resources to such inexperienced mothers. Web-based screening and communication are expected to play a significant role not only for parenting under the “new normal” lifestyle due to the SARS-CoV-2 pandemic but also for parents who do not prefer face-to-face communication.

The Japanese government supports the Society 5.0 project, which aims to integrate cyber and physical spaces (also known as the cyber-physical space) where data science can contribute to task executions and coordination [12]. In this project, we created a web-based screening system linked to an app that calculates gestational age and provides parenting information services. This system enabled researchers to collect data from mothers in various parenting environments across Japan without requiring hospital visits. However, the real-world data obtained by this system were heterogeneous. Machine learning is considered more suitable than model-driven conventional statistical methods for classifying characteristics and developing interventions based on complex data, even with a small sample size [13]. Especially in mental health research, machine learning is used for diagnosis, treatment support, research and development, and clinical work management. The increase in the number of studies suggests that machine learning may be useful for the detection and diagnosis of mental health conditions such as depression, schizophrenia, and Alzheimer disease [14]. A systematic review screened 482 papers and evaluated 11 papers that used machine learning to predict postpartum depression; it found that although a solid conclusion was not achieved since the algorithms and data sets used were heterogeneous, all studies reached an area under the curve greater than 0.7, indicating that predicting postpartum depression by machine learning is feasible [15]. Machine learning methods also allow the use of blood test data from before the onset of perinatal depression to identify high-risk populations who are the most in need of preventive interventions [16].

The disadvantages of machine learning include its complex algorithms and reproducibility. For example, k-means clustering is a machine learning–based algorithm that groups similar data by analyzing its structures and patterns; each data point is assigned to a cluster to minimize variance within the cluster while maximizing it between clusters. However, reproducing these clusters in a different data set can be challenging. Therefore, we used explainable k-means clustering to overcome these limitations [17].

The purpose of this study is to create a vulnerability classifier based on cross-sectional survey data on maternal resilience and adaptation and to examine the identified psychosocially vulnerable groups. This study also attempts to determine the psychosocial status of mothers during other child-rearing periods, by confirming whether the classifier can be applied to infant and toddler populations to identify similarly vulnerable groups.

## Methods

### Study Design and Setting

This study included a cross-sectional survey and machine learning stratification. Recruitment, opt-in informed consent, and data collection were conducted through a web application that rewarded participants with the equivalent cost of the internet resources used to complete the survey. We used a Japanese web service company called Milcare to conduct the survey. For those who downloaded Milcare’s app, which is used to record the number of weeks of pregnancy, an advertisement for the survey was shown in the app for the duration of our survey setting. The system was designed to provide survey participants incentives such as gift cards and childcare goods through the app. The app was available to Japanese smartphones and had been downloaded approximately 5000 times at the time of the survey. All data reported by the participants in the web-based survey were included as outcomes. The response period was from January to June 2020. A maternal personality traits classifier was created from the newborn cohort using explainable k-means clustering. The classifier was then applied to the infant cohort and the toddler cohort, and the features of each group were analyzed.

### Participants

The target population of this study was parenting mothers with newborns to 2-year-old toddlers. The eligibility criteria for the participants were (1) mothers with children aged <2 years; (2) web-based consent to participate in the survey through the Milcare web service; (3) ability to read, write, and understand Japanese while using the internet; and (4) ability to complete a questionnaire using a smartphone. The exclusion criteria were those who (1) completed the survey in less than 8 minutes (the minimum time required to read and answer all questions) and (2) abandoned the survey during the process.

### Ethical Considerations

Ethical review approval was obtained because this study involves human subjects: Osaka University Hospital Observational Research Ethics Review Committee (19290-2) and Research Ethics Review Committee of RIKEN (2020-23(2)). Data for the study were analyzed anonymously and no individuals were identified. In addition, consent for primary data acquisition and secondary use was obtained.

### Patient and Public Involvement

Although some of the researchers have parenting experience, neither patients with depressive moods nor the public were involved in the design, planning, conduct, or reporting of this study.

### Assessment

The mothers’ resilience and adaptation were assessed using the CPRA. The CPRA consisted of 5 domains (child’s temperament and health, environmental resources, perceived support, mother’s cognitive and behavioral characteristics, and psychological adaptation to parenting) and 21 subscales (Table 1). Responses were collected on a 5-point Likert scale with higher scores indicating increased parenting difficulty [8].

Depressive mood was assessed using the Edinburgh Postnatal Depression Scale (EPDS). In Japan, an EPDS score of 9 or above is used as the cutoff for clinical screening, which has a sensitivity of 75% to 82% and a specificity of 93% to 95% for high-risk depression [18-20]. Therefore, we defined an EPDS score of 9 or above to indicate depressive mood in this study. The Sense of Coherence (SOC) scale was used to assess how one understands, manages, and feels emotional meaning when experiencing stress [21]. The Postpartum Bonding Questionnaire (PBQ) was used to assess mother-infant bonding, with a high score (≥13) indicating impaired bonding [22]. The Pittsburgh Sleep Quality Index (PSQI) was used to assess sleep quality, where a high score (≥5.5) indicates poor sleep quality [23,24].

**Table 1:** Comprehensive Scale for Parenting Resilience and Adaptation domains and subscales.

Domain	Abbreviation	Subscale
Child’s temperament and health	Child	Child’s temperament and health
Environmental resources	Environment	Child-rearing or long-term care burden Parental autonomy Partner autonomy Partner temperament Relationship with the medical staff
Perceived support	Support	Husband’s or partner’s support Lack of psychological support from husband or partner Parental support Sufficient social support
Mother’s cognitive and behavioral characteristics	Cognitive	Attachment problems Emotional control Inattentiveness Simultaneous or overall processing Social intolerance Systemization urge
Psychological adaptation to parenting	Psychological	Lack of self-confidence Possibility of coping Love for the child Self-esteem Self-responsibility

### Explainable Clustering With Decision Tree

To classify the characteristics of resilience and adaptation difficulties, a clustering algorithm, explainable k-means clustering, was used. Explainable k-means clustering is an unsupervised clustering algorithm, characterized by its use of decision trees with minimum leaf size for data set partitioning. These trees are meticulously designed to contain a constrained number of nodes and leaves, adhering to the principle of parsimony. Specifically, the number of leaves is deliberately set to correspond with the desired number of clusters (k) in the k-means algorithm. This strategic design enhances both the interpretability and comprehensibility of the clustering process. Consequently, this framework facilitates the application of classifiers to external populations, provided that these populations are quantified on analogous scales. Such an approach underscores the algorithm’s use in ensuring coherent and interpretable clustering outcomes, which are pivotal for the analysis of heterogeneous data sets [17]. For estimating the number of groups, gap statistics analysis was conducted using the CPRA data set from the newborn cohort. Gap statistics analysis compares the change in cluster dispersion with that expected under an acceptable, reference null distribution using the output of any clustering algorithm [25]. Using these results, participants in the newborn cohort were stratified into groups using an unsupervised explainable k-means algorithm with a decision tree, depending on the 5 CPRA domains. Last, the clustering-based anomaly detection algorithm, the so-called decision tree, was applied to the infant cohort and the toddler cohort.

### Comparison of Mothers’ States in the Identified Groups

We described the characteristics of the CPRA domain scores and the psychological assessment scores (EPDS, PBQ, and PSQI) for each group. The stratification of groups in each cohort was plotted and the mean score of the 21 CPRA subscales for each of the 5 stratified groups was calculated and plotted as a radar chart.

### Statistical Analysis

We analyzed the characteristics of the stratified groups in each cohort, using the group with no difficulties as a reference. The prevalence of depressive moods (EPDS score≥9), bonding problems (PBQ score≥13), and sleep problems (PSQI score≥5.5) was used for logistic regression analysis with relative prevalence (RP), 95% CI, and *P* value. The distributions of the CPRA and psychosocial scores of the stratified groups in the cohorts were compared using the Kruskal-Wallis test. The results of the Kolmogorov-Smirnov test showed deviations from the normal distribution for many variables, and the Levene test may have violated the assumption of equal variability in the EPDS and PBQ (Multimedia Appendix 1). Therefore, the Kruskal-Wallis test was performed as a multiple-group comparison.

All data from participants who completed the survey within the specified time frame were included in the analysis. Participants’ characteristics were expressed using descriptive statistics, and *P*<.05 was considered statistically significant.

All statistical analyses were performed using R (version 3.6.2; R Foundation for Statistical Computing). We used the *ExKMC* package to produce explainable k-means clustering and the *ggstatsplot* and *ggplot* packages for data visualization, all of which are available on GitHub (GitHub Inc) [17,26]. The *ExKMC* package automatically produces the shallowest tree depth.

## Results

### Participant Characteristics

**Figure 1:**
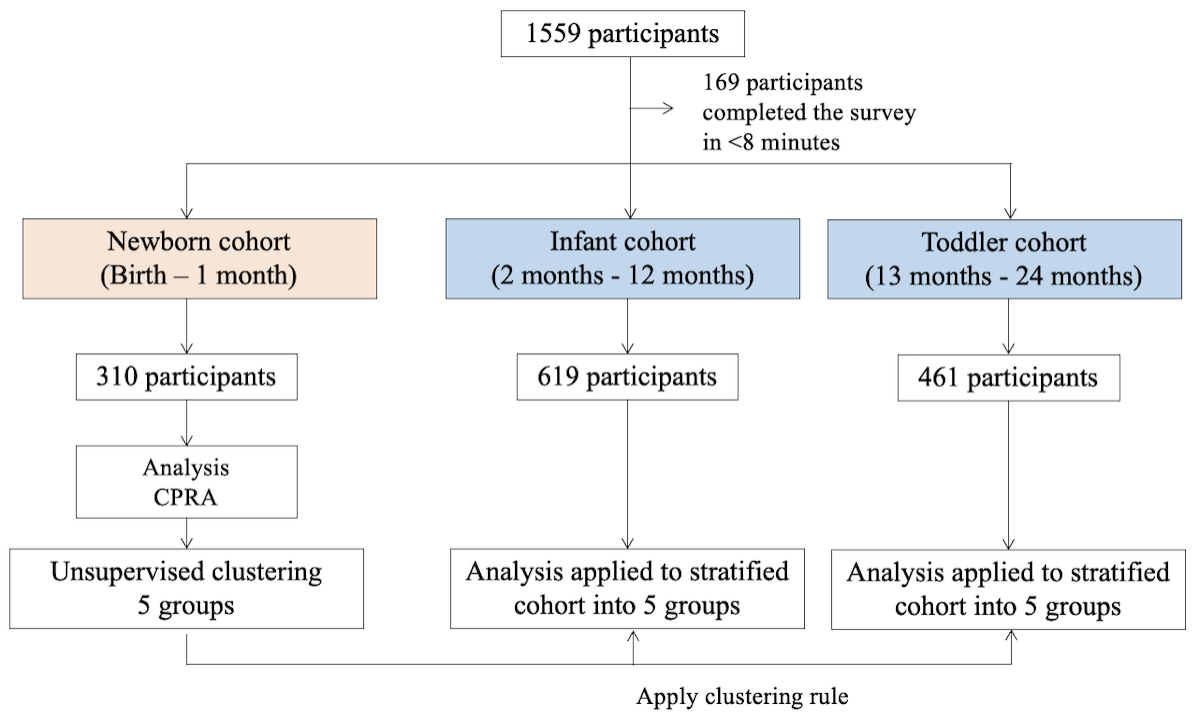
Study flow. CPRA: Comprehensive Scale for Parenting Resilience and Adaptation.

From the web-based recruitment, 1559 participants completed the web survey. Data from those who took at least the minimum required time to read and answer the questions were analyzed. The participants were divided into the newborn cohort (n=310; mean age 31, SD 4.60 years), infant cohort (n=619; mean age 31.5, SD 4.15 years), and toddler cohort (n=461; mean age 32.1, SD 4.16 years). The newborn cohort was analyzed to generate a classifier, and the classifier was applied to the infant cohort and toddler cohort (Figure 1). The CPRA domain scores and the psychosocial assessments (EPDS, PBQ, and PSQI) in each cohort are represented in Table 2.

Overall, parenting difficulties as assessed by the CPRA tended to be more severe during the newborn period, which is up to 1 month post partum (Table 2). Statistically significant differences were observed in the environmental and psychological domains of the CPRA, as well as for the EPDS, PBQ, and PSQI (all *P*<.001).

**Table 2:** Comprehensive Scale for Parenting Resilience and Adaptation and psychosocial scores by cohort.

Scores	Newborn (n=310), median (IQR)	Infant (n=619), median (IQR)	Toddler (n=461), median (IQR)	*P* value^a^
Child	2.20 (1.80-2.80)	2.20 (1.80-2.60)	2.20 (1.80-2.60)	.005
Cognitive	2.36 (2.00-2.71)	2.29 (1.93-2.64)	2.36 (2.00-2.71)	.01
Environment	2.35 (2.05-2.75)	2.25 (1.95-2.55)	2.25 (2.00-2.60)	<.001
Psychological	2.47 (2.05-2.95)	2.26 (1.95-2.74)	2.42 (2.05-2.79)	<.001
Support	2.47 (1.88-2.93)	2.27 (1.80-2.73)	2.40 (2.00-2.80)	.003
EPDS^b^	9 (5-13)	5 (2-9)	6 (3-10)	<.001
PBQ^c^	10 (6-18)	9 (6-14)	12 (7-17)	<.001
PSQI^d^	7 (5-9)	7 (5-9)	5 (4-8)	<.001

^a^Kruskal-Wallis rank sum test.

^b^EPDS: Edinburgh Postnatal Depression Scale.

^c^PBQ: Postpartum Bonding Questionnaire.

^d^PSQI: Pittsburgh Sleep Quality Index.

### Explainable Clustering

**Figure 2:**
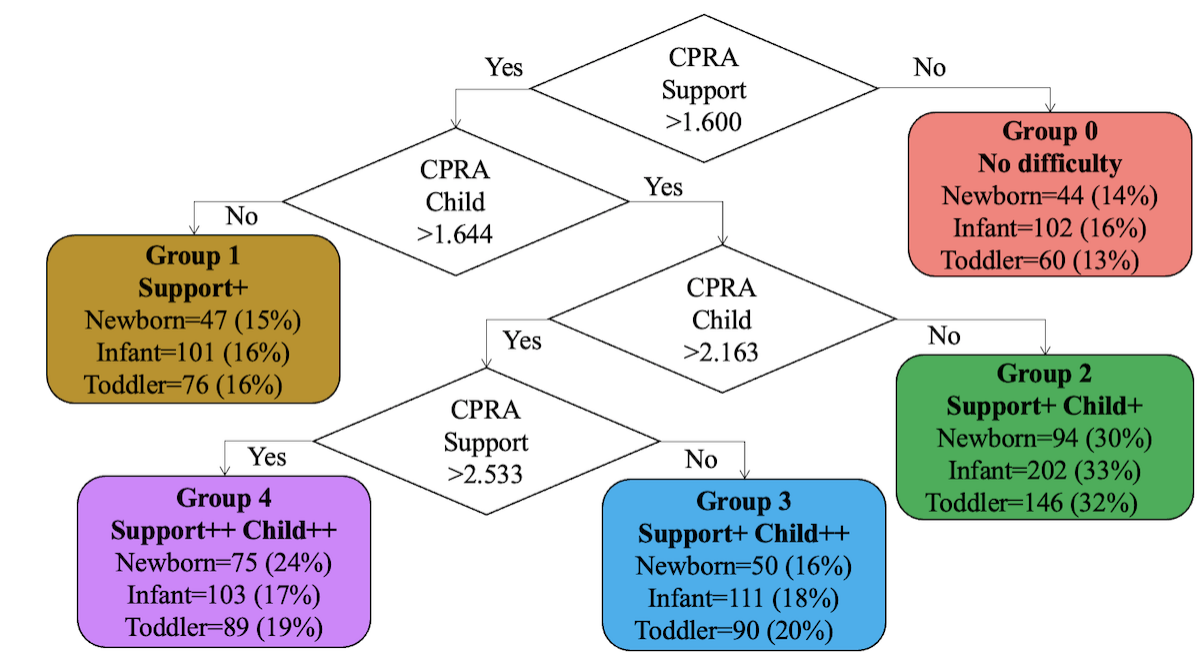
Decision tree explanation for k-means clustering. CPRA: Comprehensive Scale for Parenting Resilience and Adaptation.

**Figure 3:**
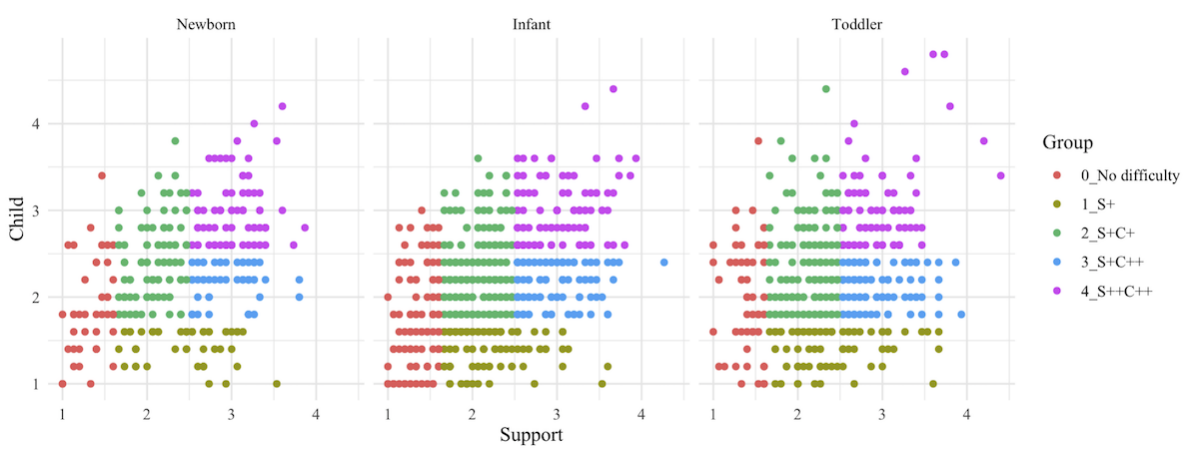
Comprehensive Scale for Parenting Resilience and Adaptation domains scores (child's temperament [C] and perceived support [S]) in the 3 cohorts (newborn, infant, and toddler), colored by the stratified groups.

**Figure 4:**
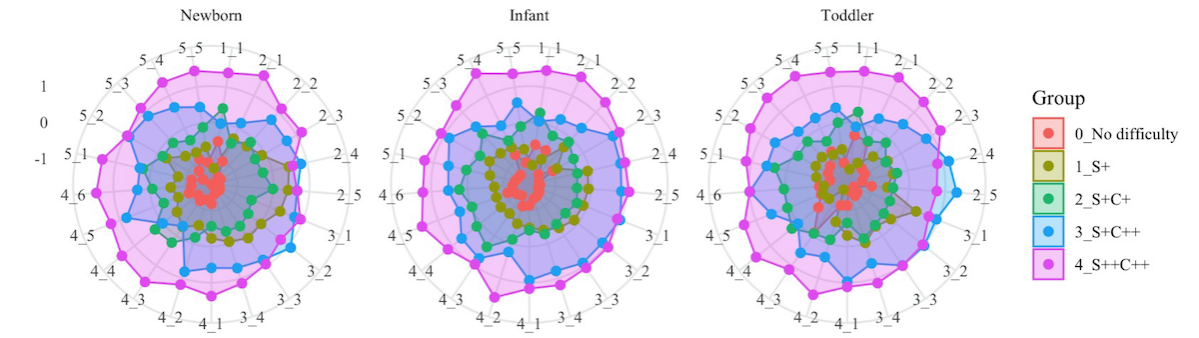
Comprehensive Scale for Parenting Resilience and Adaptation scores for 21 factors in the groups. C: child's temperament; S: perceived support.

Using gap analysis to decide the optimal number of the clustering revealed that 5 groups were suitable for k-means clustering (grouping) of the participants based on the similarities in CPRA scores. Using the explainable k-means method, the participants in the newborn cohort were stratified into 5 groups based on multivariate factors, and a decision tree explaining how to stratify these groups was generated using 2 evaluation axes: perceived support–related and child’s temperament–related difficulties (Figure 2). Using the classifier as a decision tree algorithm, 5 groups were stratified in each of the 3 cohorts. According to the classifier rules, Group 0 represented those with no perceived support–related difficulties, Group 1 represented those with moderate perceived support–related difficulties (Support+), Group 2 represented those with moderate perceived support–related difficulties and child’s temperament–related difficulties (Support+Child+), Group 3 represented those with moderate perceived support–related difficulties and severe child’s temperament–related difficulties (Support+Child++), and Group 4 represented those with severe perceived support–related and child’s temperament–related difficulties (Support++Child++; Figure 2). A split of similar proportions was achieved, with the maximum difference of 5% occurring in Group 4. To illustrate the distribution of the stratified groups in each cohort, we focused on the child and support scores, which were used as features for classifier generation; plotted them on the vertical and horizontal axes, respectively; and colored each group to visualize them (Figure 3). The CPRA scores for each cohort were concentrated at 2-3 points, but the distribution of outliers was different for each cohort (Figure 3). The 21 subscales of the CPRA for each stratified group in each cohort were plotted in a radar chart (Figure 4).

### Regression Analysis and Characteristics of the Detected Groups

**Figure 5:**
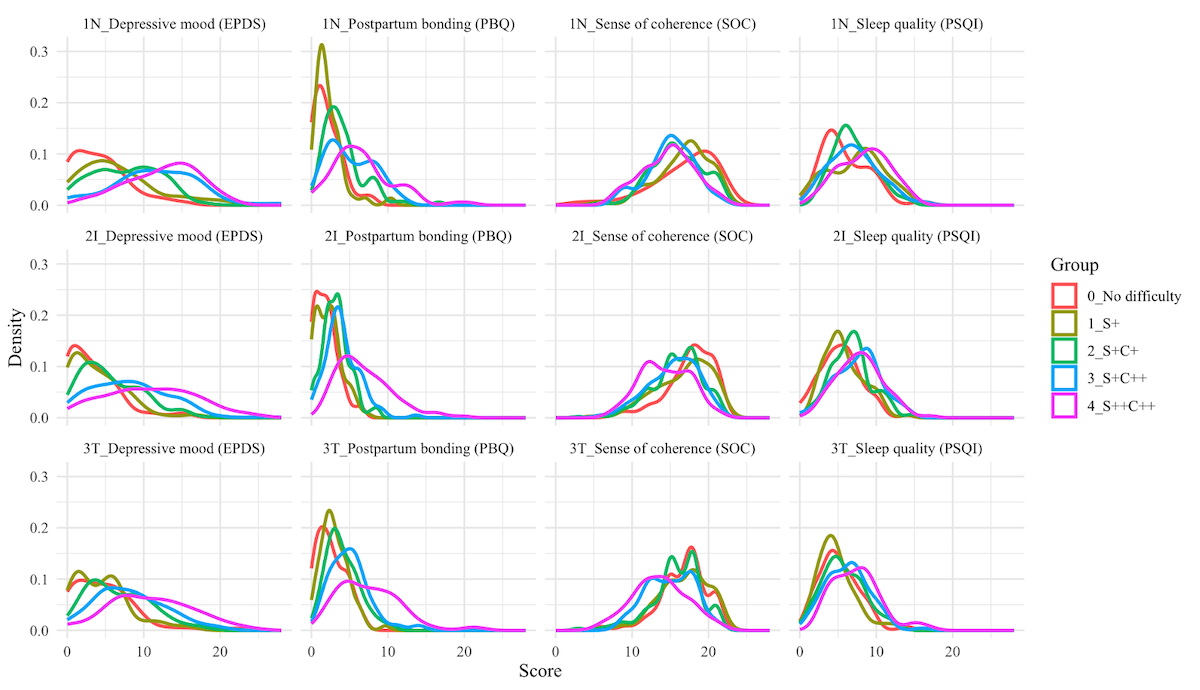
Mothers’ condition in the stratified groups. Postnatal depressive mood was assessed using the EPDS, postpartum bonding was assessed using the PBQ, sense of coherence was assessed using the SOC, and sleep quality was assessed using the PSQI. 1N: newborn cohort; 2I: infant cohort; 3T toddler cohort; C: child's temperament; EPDS: Edinburgh Postnatal Depression Scale; PBQ: Postpartum Bonding Questionnaire; PSQI: Pittsburgh Sleep Quality Index; S: perceived support; SOC: Sense of Coherence.

Psychosocial conditions in each cohort were plotted as density plots for the EPDS, PBQ, SOC, and PQSI scores, colored according to the stratified groups (Figure 5). In the newborn cohort (n=310), Group 4 (Support++Child++) was the group with the greatest difficulties, having higher incidences of problems with depressed mood (RP 5.87, 95% CI 2.77-12.45), bonding (RP 5.38, 95% CI 2.53-11.45), and sleep quality (RP 1.70, 95% CI 1.20-2.40) compared to the group with no difficulties in perceived support (Group 1; Table 3). In the infant cohort (n=619), the stratified group with the greatest difficulties had higher incidences of problems compared to the group with no difficulties, with depressed mood (RP 9.05, 95% CI 4.36-18.80) and sleep quality (RP 8.69, 95% CI 4.62-16.37) having a greater RP than the newborn cohort, but a smaller RP for bonding (RP 1.63, 95% CI 1.29-2.06; Table 3). In the toddler cohort (n=461), the stratified group with the greatest difficulties had higher incidences of problems with depressed mood (RP 4.63, 95% CI 2.38-9.02), bonding (RP 3.19, 95% CI 2.03-5.01), and sleep quality (RP 1.72, 95% CI 1.23-2.42) compared to the group with no difficulties.

**Table 3:** Comparison of the frequency of problems with depressive mood, postpartum bonding, and sleep quality.

Cohort and group			Depressive mood (EPDS^a^≥9)						Postpartum bonding (PBQ^b^≥9)							Sleep quality (PSQI^c^≥5.5)				
			Count, n (%)		RP^d^ (95% CI)		*P* value		Count, n (%)		RP (95% CI)		*P* value		Count, n (%)			RP (95% CI)		*P* value
**Newborn cohort (n=310)**																				
	0 (no difficulties; n=44)	6 (14)		N/A^e^		N/A		6 (14)		N/A		N/A		20 (45)			N/A		N/A	
	1 (S^f^+; n=47)	15 (32)		2.34 (1.00-5.49)		.05		5 (11)		0.78 (0.26-2.38)		.66		30 (64)			1.47 (1.00-2.15)		.05	
	2 (S+C^g^+; n=94	43 (46)		3.35 (1.54-7.29)		.002		35 (37)		2.73 (1.24-6.01)		.01		66 (70)			1.58 (1.11-2.24)		.01	
	3 (S+C++; n=50)	37 (74)		5.43 (2.53-1.62)		<.001		26 (52)		3.81 (1.73-8.40)		.001		34 (68)			1.53 (1.05-2.22)		.03	
	4 (S++C++; n=75)	60 (80)		5.87 (2.77-12.45)		<.001		55 (73)		5.38 (2.53-11.45)		<.001		58 (77)			1.70 (1.20-2.40)		.003	
**Infant cohort (n=619)**																				
	0 (no difficulties; n=102)	7 (6.9)		N/A		N/A		9 (8.8)		N/A		N/A		47 (46.1)			N/A		N/A	
	1 (S+; n=101)	10 (9.9)		1.44 (0.57-3.64)		.44		14 (13.8)		1.57 (0.71-3.46)		.26		44 (43.6)			0.97 (0.71-1.30)		.82	
	2 (S+C+; n=202)	50 (24.8)		3.61 (1.70-7.67)		<.001		46 (22.7)		2.58 (1.32-5.06)		.006		135 (66.8)			1.45 (1.15-1.82)		<001	
	3 (S+C++; n=111)	50 (45.0)		6.56 (3.12-13.81)		<.001		41 (36.9)		4.19 (2.14-8.18)		<.001		80 (72.1)			1.56 (1.23-1.98)		<.001	
	4 (S++C++; n=103)	64 (62.1)		9.05 (4.36-18.80)		<.001		79 (76.7)		8.69 (4.62-16.37)		<.001		75 (72.8)			1.63 (1.29-2.06)		<.001	
**Toddler cohort (n=461)**																				
	0 (no difficulties; n=60)	8 (13)		N/A		N/A		15 (25)		N/A		N/A		24 (40)			N/A		N/A	
	1 (S+; n=76)	8 (11)		0.79 (0.31-1.98)		.61		19 (25)		1.00 (0.56-1.80)		.99		22 (29)			0.73 (0.46-1.17)		.19	
	2 (S+C+; n=146)	50 (34.2)		2.57 (1.30-5.09)		.007		62 (42.5)		1.70 (1.05-2.74)		.03		67 (45.9)			1.17 (0.82-1.67)		.38	
	3 (S+C++; n=90)	37 (41)		3.08 (1.55-6.15)		.001		54 (60)		2.40 (1.50-3.84)		<.001		53 (59)			1.49 (1.04-2.12)		.03	
	4 (S++C++; n=89)	55 (62)		4.63 (2.38-9.02)		<.001		71 (80)		3.19 (2.03-5.01)		<.001		60 (67)			1.72 (1.23-2.42)		.002	

^a^EPDS: Edinburgh Postnatal Depression Scale.

^b^PBQ: Postpartum Bonding Questionnaire.

^c^PSQI: Pittsburgh Sleep Quality Index.

^d^RP: relative prevalence (compared with Group 0).

^e^N/A: not applicable.

^f^S: perceived support.

^g^C: child’s temperament.

## Discussion

### Principal Findings

The classifier based on the resilience and adaptation assessment (CPRA) stratified the mothers in the newborn cohort, the start-up stage of childcare, into 5 groups and identified vulnerable psychosocial groups. The score of resilience to the child’s temperament and perceived support was selected as an important feature for building an explainable classifier, instead of environmental resources, the mother’s cognitive-behavioral characteristics, and psychological adaptation to parenting. Depending on the decision tree–based explainable classifier, psychosocially vulnerable groups were also identified in the qualitatively different infant and toddler cohorts. The identified group with high sensitivity to the temperament of their children and difficulty in perceiving support showed high RP for depressed mood and poor sleep quality, especially in the infant cohort. This group was particularly vulnerable to severe psychosocial problems in the infant cohort.

### Limitations

The study data were collected using a web-based self-reported survey to acquire large-scale nationwide data. Therefore, the degree of objectivity, the participants’ backgrounds, and family characteristics (ie, the partner’s housework load and the child’s developmental disabilities) were not guaranteed. The possibility that false responses were treated as true values cannot be excluded. Nevertheless, a web-based survey method with limited psychological barriers was chosen because it increased the ease of participation, so that even mothers who are too busy to make time for a formal interview or have difficulties with interpersonal conversations could be represented. This study saw a greater prevalence of perinatal depressive mood (EPDS>9; 480/1390, 34.5%) than that reported by a previous face-to-face Japanese study (15,506/108,431, 14.3%). This disparity may be due to the anonymity of the survey environment, which allowed mothers to share grievances beyond supporting the image of a good, tolerant mother. We are conducting a face-to-face observational study and analyzing the target population gap caused by the data collection environment, as the question of whether web-based or in-person data accurately reflect the true state of a participant must be considered in the context of increasing web-based communication to minimize error.

The lack of background information also limits the analysis of individual factors in this study. Environmental variables could have a significant impact on the mother’s family environment, but the CPRA could not assess “home-town delivery,” a common practice in Japan where mothers return to their parent’s homes before and after childbirth. Future research using the CPRA with more detailed data on individual characteristics, such as the home environment or family structure, will be needed. In addition, those who had difficulty perceiving support and were sensitive to their child’s temperament are particularly prone to serious psychosocial problems in the infant cohort, but it is difficult to determine whether this is a bias in the cross-sectional survey population or whether those with resilience difficulties are more specific to the infant period. Therefore, we are conducting a longitudinal survey with the environmental information to examine the effects of the environmental setting or the time period.

The accuracy of the clustering algorithm will be limited because of the nature of this algorithm, with a trade-off between explainability and accuracy [17]. In addition, no general method of predetermining k is established [27]. Although the EPDS, PBQ, SOC, and PSQI scores were significantly different across the 5 groups, especially the groups with no versus greatest difficulties in resilience and adaptation, we believe that further investigation is needed to use the partition score as a clear cut-off point for detecting persons with psychosocial disabilities.

### Comparison With Prior Work

Previous studies have reported that personality traits can influence perinatal depressive mood. For example, a study involving 15,012 mothers, including 13.1% with depressive moods (an EPDS score≥9 in 1 month was defined as “postpartum depressive symptoms” in that study), indicated that increased neuroticism and reduced extraversion were associated with postpartum depressive symptoms [28]. Regarding interventions for perceived support or parenting resources, a review showed that online peer support groups offered informational and emotional support and positively impacted maternal mental health [27]. A path analysis of web-based survey data collected from Japanese parents of children aged 3-5 years revealed that childcare support had no direct positive effect on children’s social development; however, the benefits of childcare support were mediated by its impact on parents’ psychological state and parenting style, which improved child social development [29]. This indicates that supporting mothers, according to their adaptive tendencies, may positively affect their children’s development. A study of 398 Australian women from the prenatal period to 1 year post partum suggested that the dual intervention of social support and the recognition of prenatal depressive symptoms is a promising strategy to prevent persistent depressive symptoms [30]. This is because digital technology supports the core values associated with psychosocial intervention and fulfills the “ancillary values” that constrain how coproduction operates [31]. To assess the effects of such web-based psychosocial intervention for mothers, we are conducting a randomized controlled trial of a web-based screening and feedback program using the same assessment instruments.

Our study was conducted before and after April 2020, and although the pandemic may have had an impact, we did not detect any significant differences when comparing the different survey periods (Multimedia Appendix 2). This may be because the study was completed in June 2020; however, the data set collected from 5 countries during the COVID-19 pandemic from July to December 2020 also showed that the symptoms of maternal depression and anxiety can be predicted using machine learning algorithms and that efficient tools can be used to predict maternal depression and anxiety [32].

### Conclusions

The classifier, generated from the data from the most stressful and confusing period (newborn cohort) using an unsupervised clustering algorithm with enhanced explanatory and applicability, could be adapted to another parenting cohort (infant and toddler cohort), and psychosocially vulnerable groups were detected. Mothers with high sensitivity to the temperament of their children and difficulties in perceiving support would be prone to depressed mood and poor sleep quality, especially in the infant cohort. To overcome the study’s limitations, further research in other study designs is needed. Considering the additional research, the classifier will help support child-rearing in the Society 5.0 era according to the resilience and adaptation characteristics of individual mothers and is likely to contribute to the implementation of web-based child-rearing support.

## Appendix

Multimedia Appendix 1 Results of Kolmogorov-Smirnov test and Levene test.

Multimedia Appendix 2 Comparison of psychological measures before and during pandemic.

## Abbreviations

CPRA: Comprehensive Scale for Parenting Resilience and Adaptation
EPDS: Edinburgh Postnatal Depression Scale
PBQ: Postpartum Bonding Questionnaire
PSQI: Pittsburgh Sleep Quality Index
RP: relative prevalence
SOC: Sense of Coherence

## Data Availability

The data sets generated and analyzed during this study are available from the corresponding author on reasonable request.
