# Supplementary material for: Explainable Machine Learning Classification to Identify Vulnerable Groups Among Parenting Mothers: Web-Based Cross-Sectional Questionnaire Study
Source: JMIR Form Res. 2024 Feb 7;8:e47372. doi: 10.2196/47372 (PMC10882468; doi:10.2196/47372)
Supplement: Multimedia Appendix 1 [file formative_v8i1e47372_app1.docx]

**Multimedia Appendix 1. Results of Kolmogorov-Smirnov test and Levene test**

Kolmogorov-Smirnov test

Child

Newborn: P = 0.0146

Infant: P = 0.0004

Toddler: P = 0.0003

Cognitive

Newborn: P = 0.2000

Infant: P = 0.0995

Toddler: P = 0.1371

Environment

Newborn: P = 0.4796

Infant: P = 0.3739

Toddler: P = 0.0345

Psychological

Newborn: P = 0.0262

Infant: P = 0.1452

Toddler: P = 0.6692

Support

Newborn: P = 0.1121

Infant: P = 0.0310

Toddler: P = 0.6726

EPDS

Newborn: P = 0.0275

Infant: P < 0.0001

Toddler: P = 0.0002

PBQ

Newborn: P = 0.00002

Infant: P < 0.0001

Toddler: P = 0.00002

PSQI

Newborn: P = 0.0059

Infant: P = 0.0005

Toddler: P < 0.0001

Levene test

Child: P = 0.3563

Cognitive: P = 0.8377

Environment: P = 0.0307

Psychological: P = 0.1454

Support: P = 0.2286

EPDS: P = 0.0098

PBQ: P = 0.0006

PSQI: P = 0.0833
