# Supplementary material for: Explainable Machine Learning Classification to Identify Vulnerable Groups Among Parenting Mothers: Web-Based Cross-Sectional Questionnaire Study
Source: JMIR Form Res. 2024 Feb 7;8:e47372. doi: 10.2196/47372 (PMC10882468; doi:10.2196/47372)
Supplement: Multimedia Appendix 2 [file formative_v8i1e47372_app2.docx]

**Multimedia Appendix 2. Comparison of Before/During Pandemic**

|  | **Before Pandemic**  N = 706 | **During Pandemic**  N = 684 | **p-value***^2^* |
| --- | --- | --- | --- |
| Child | 2.20 (1.80, 2.60) | 2.20 (1.80, 2.60) | 0.4 |
| Cognitive | 2.29 (2.00, 2.71) | 2.36 (2.00, 2.71) | 0.3 |
| Environment | 2.30 (2.00, 2.60) | 2.25 (2.00, 2.60) | 0.3 |
| Psychological | 2.37 (2.00, 2.79) | 2.37 (2.00, 2.84) | 0.6 |
| Support | 2.40 (1.87, 2.80) | 2.27 (1.87, 2.87) | 0.4 |
| EPDS | 6 (3, 11) | 6 (3, 10) | 0.5 |
| PBQ | 10 (6, 16) | 10 (6, 16) | >0.9 |
| PSQI | 6 (4, 9) | 6 (4, 8) | 0.052 |
| *^1^* Median (IQR); n (%) | | | |
| *^2^* Wilcoxon rank sum test | | | |
